# A Case of Severe Accidental Hypothermia Successfully Treated with Cardiopulmonary Bypass

**DOI:** 10.5811/cpcem.2016.11.32919

**Published:** 2017-01-18

**Authors:** Erfun M. Hatam, Andrew Cameron, Dimitri Petsikas, David Messenger, Ian M. Ball

**Affiliations:** * Western University, Schulich School of Medicine and Dentistry, London, Ontario, Canada; † University of Toronto, Department of Emergency Medicine, Toronto, Ontario, Canada; ‡ Queen’s University, Kingston General Hospital, Division of Cardiac Surgery, Kingston, Ontario, Canada; § Queen’s University, Department of Emergency Medicine & Critical Care Medicine, Kingston, Ontario, Canada; ¶ Western University, Department of Epidemiology and Biostatistics, Division of Critical Care Medicine, London, Ontario, Canada

## Abstract

After missing for seven days, a 34-year-old female was found with a rectal temperature of 19.8oC. Instead of attempting aggressive rewarming in the emergency department she was directly transferred to the operating room for extracorporeal rewarming. She received cardiopulmonary bypass (CPB) for 66 minutes at an initial warming rate of 12oC/ hour and warmed to 36.2oC. Her postoperative course was complicated by sepsis, which eventually led to bilateral below-knee amputations after refusing antibiotics. She was discharged 22 days after admission, with full neurologic recovery. This remarkable case highlights the emerging role of CPB as the definitive therapy for severe accidental hypothermia.

## INTRODUCTION

Hypothermia is defined as a decrease in core body temperature, broadly classified as mild (32–35 °C), moderate (28–32 °C), and severe (< 28 °C).^1^ The spectrum of treatments is equally broad. At the mild end of the spectrum, peripheral rewarming strategies such as the application of warming packs to the groin and axillae are adequate. However, severe hypothermia requires invasive central rewarming interventions.^2^ This case report features a patient who presented with severe accidental hypothermia and her ensuing management using cardiopulmonary bypass.

## CASE PRESENTATION

A 34-year-old female with a history of intravenous drug use and recent suicidal ideation was reported missing by her family in late winter. She was missing for a total of seven days before she was located by the police. During this time the outdoor temperature ranged from −10.4 to +1.2°C. She was found outdoors in a remote, wooded area and was transported by air to a tertiary care center.

Upon the paramedics arrival to the scene the patient was unresponsive with a Glasgow Coma Scale (GCS) of 4. Her pupils were 7 mm and non-responsive. She was severely hypothermic, with a rectal temperature of approximately 23°C. She had a heart rate of 30 beats per minute, respiratory rate of 6 breaths per minute, and an unmeasurable blood pressure and pulse oxygen saturation. Peripheral pulses were not palpable in her limbs. Her extremities were diffusely cyanotic and had delayed capillary refill. Her legs were edematous and blistered bilaterally. Her mouth opening was 1 cm, and she bit down at any attempts to introduce an oral airway. Paramedics applied warming packs to her axillae and groin, obtained intravenous (IV) access and initiated fluid resuscitation with 1000 mL normal saline in an 18-gauge peripheral IV line at 75 mL/hr.

She was transported by rotary wing air ambulance from the scene to a tertiary care Level 1 trauma center. On presentation to the emergency department (ED), she was identified to have a GCS of 3, with a heart rate of 40 beats per minute, respiratory rate of 8 breaths per minute, an unmeasurable blood pressure and pulse oxygen saturation, and a rectal temperature of 19.8 °C. Her cardiac rhythm showed sinus bradycardia. The patient’s airway was secured by rapid sequence intubation with 2 mg midazolam, and 30 mg rocuronium. A femoral central venous catheter was inserted, and she continued to receive IV fluids in the form of two liters of Lactated Ringer’s warmed to 41 °C through a femoral 3-lumen central line. A forced air rewarming unit was also applied. The patient was not observed to have any episodes of cardiac dysrhythmia during her time in the ED.

Rather than initiating invasive rewarming in the ED, in consultation with the cardiac surgeon a decision was made to transfer the patient directly to the operating room (OR) for CPB for the purpose of rewarming. The patient was transferred to the OR 40 minutes after presenting to the ED. At the time of transfer, the patient’s rectal temperature was 20.2 °C. In the OR, the patient had a junctional escape rhythm of 50 beats per minute. Because of severe stocking-like erythema and edema in her lower extremities, IV fluid bags were placed under her feet to prevent ulcer development during the operation. She underwent a median sternotomy, revealing a dilated heart with a soft ascending aorta. She was cannulated with a two-stage venous cannula via atrial appendage, infused with heparin, and rewarmed at a rate of 12°C per hour. The total OR time was 3 hours and 45 minutes, with 66 minutes spent on CPB. Upon transfer from the OR the patient had been rewarmed to a temperature of 36.9 °C as seen in Image. The patient was noted to be coagulopathic secondary to hypothermia and hemodilution, and therefore was administered protamine, fresh frozen plasma, platelets, and recombinant factor VIIa to reduce postoperative bleeding.

The patient was subsequently transferred to the intensive care unit. On post-operative day 5 she developed *Klebsiella oxytoca* and *Enterococcus fecalis* bacteremia and sepsis, attributed to severe skin and soft tissue infection of her bilateral lower extremities. This was initially treated with Piperacillin and Tazobactam. The patient was seen by plastic surgery for wound care. Her blisters and necrotic tissue were debrided and sterile moist dressing changes were performed daily. The patient was initially extubated and then re-intubated on post-operative day 7 because of increased oxygen requirements. Evidence of necrosis was noted in her toes on post-operative day 7. At this point her antibiotics were changed to imipenem and vancomycin. The patient was successfully extubated on post-operative day 13.

The consultant vascular surgeon recommended staged amputations of both lower extremities. Unfortunately, the patient was non-compliant with IV antibiotics and did not consent to surgery. The patient was assessed by psychiatry and found to be emotionally distant with a depressed mood but with no evidence of psychosis or delirium. She was deemed to be competent in her decisions. She was also offered addiction counselling but refused. Although the patient ultimately accepted oral antibiotic therapy, the condition of her lower extremities continued to worsen, and she eventually developed frank necrosis and dry gangrene. The patient expressed interest in hyperbaric oxygen treatment, and on post-operative day 39 she was transferred to another tertiary care center for foot-preserving therapy. Unfortunately, conservative measures and hyperbaric treatment were unsuccessful and she ultimately consented to bilateral below-knee amputations, which took place 84 days after her initial presentation. Despite a total of up to seven days of severe accidental hypothermia, the treatments outlined above allowed the patient to make a complete neurologic recovery.

## DISCUSSION

The patient in this case presented with a rectal temperature of 19.8 °C, meeting criteria for severe hypothermia. The risk of cardiac arrest is greatly increased with temperatures below 28 °C.^3^ This justified the use of invasive central rewarming interventions, which are reserved for the most severe cases of hypothermia. The most rigorous central rewarming strategy is CPB, which uses extra-corporeal membrane oxygenation (ECMO) to permit oxygenation, filtration, or in this case, rapid warming of blood. ECMO may be complicated by hemorrhage (it requires systemic anticoagulation) renal failure, neurologic impairment, and pulmonary edema.^4^–^6^ This case is unique since the patient survived without any of the aforementioned severe complications and made a complete neurologic recovery.

Most cases that involve ECMO for rewarming have used femoral-femoral-bypass, and report mortality rates ranging from 16–87% depending on the cause of hypothermia.^4^,^5^,^7^,^8^,^9^ The patient in this case received an open bypass by sternotomy. Sternotomy was used to access the central vessels rather than peripheral cannulation because the patient’s femoral vessels were small and cannulating them may have caused damage and sub-optimal flow rates. Another benefit of this approach was that it could allow for cardiac massage, which would lead to higher flow rates thereby allowing faster rewarming.^8^

There is lack of agreement in the literature regarding the appropriate rate approach to rewarming a severely hypothermic patient. A recent review by Brown et al. (2012) advised clinicians to consider multiple factors when selecting the rate, such as accessibility to an appropriate facility, local expertise, and patient characteristics. While CPB is certainly the fastest rewarming method at approximately 9 °C per hour, slower rates are often preferred.^2^ This reluctance may be driven by the concern for the “after drop” effect, which describes paradoxical exacerbation of the core hypothermia secondary to peripheral vasodilation and return of cold peripheral blood to the body core.^1^ Controlled hypothermia experiments have demonstrated that the after drop effect is overestimated and clinically insignificant.^11^–^13^ The patient described in this case was rewarmed at a very rapid rate of 12 °C per hour, which is 8 °C per hour faster than is typically seen in venous-venous ECMO, and 3 °C per hour faster than in standard CPB.^2^ The patient was rewarmed completely up to 36.2 °C with no after drop effect, and no other complications. Overall, there was no evidence suggesting that a faster rewarming rate was detrimental to the patient in this case.

Prehospital care of the severely hypothermic patient is critical, and the team treated the patient in this case expertly. Traditional literature advocates the avoidance of moving severely hypothermic patients brusquely, out of concern for their predilection to dysrhythmias, particularly ventricular fibrillation.^10^,^14^ These concerns are poorly supported,^1^,^15^ and it is our opinion that the prehospital team was correct to secure the patient in the transport vehicle and transport her to hospital as quickly as possible. The prehospital team was only at the site where the patient was found for five minutes before rapidly transporting her. Prompt evaluation and rapid transport by prehospital staff is what is suggested in the current literature^2^,^16^.

Another consideration when rewarming hypothermic patients is the endpoint of therapy. There is discussion regarding whether a 24-hour course of “therapeutic hypothermia” at 32–34°C is indicated in this population; however, further research is needed to provide supporting evidence.^2^ It seems appropriate to us to implement some form of targeted temperature management / therapeutic hypothermia only if there is suspicion of a concomitant anoxic insult.

Overall, effective prehospital care followed by rapid open sternotomy to allow CPB led to the survival and complete neurologic recovery of a 34-year-old woman found with a core temperature of 19.8 °C. While CPB is an effective and well-documented treatment for patients with hypothermia and cardiovascular collapse, we present a case of successful treatment of prolonged severe hypothermia with CPB in the absence of cardiovascular collapse. Many people die from accidental hypothermia every year and even more are exposed. These patients are not uniformly treated, and there is a paucity of guidelines and standards governing their treatment. This remarkable case demonstrates a viable option for treatment of such patients, and can be used to guide the development of future protocols.

## Figures and Tables

**Image:**
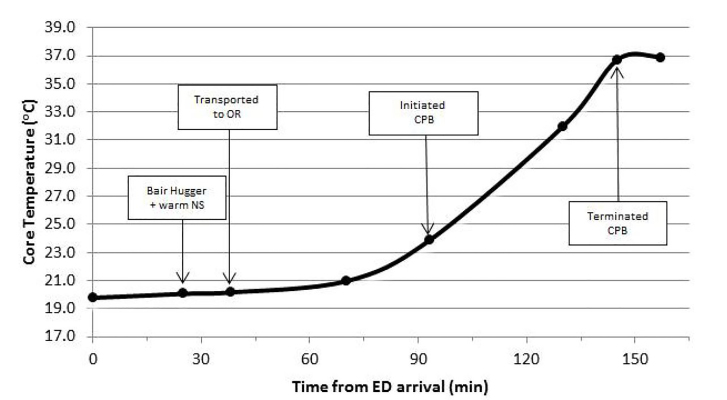
Patient’s core body temperature measurements in relation to time from arrival to the emergency department. Major intervention points have been outlined. NS=normal saline, CPB=cardiopulmonary bypass. *NS,* normal saline; *CPB*, cardiopulmonary bypass, *OR*, operating room, *ED*, emergency department

**Table:** Blood sample measurements in relation to time of presentation to the ED and core body temperature.

Lab Parameter	38min 20.2°C	70min 21.0°C	Begin CPB ↓ 93min 23.9°C	130min 32.0°C	145min 36.7°C	End CPB ↓ 157min 36.9°C	In ICU ↓ 285min -	660min -
K^+^ (mmol/L)	4.0	3.0	3.0	3.1	4.1	4.1	4.7	4.9
Na^+^(mmol/L)	140	141	138	142	141	143	140	142
pH	7.08	7.16	7.12	7.32	7.36	7.39	7.42	7.43
pCO_2_ (kPa)	59	35	-	29.8	28	29.9	33	35
pO_2_ (kPa)	60	99	-	275	409	409	251	136
HCO_3_ (mmol/L)	17	12	13	15.1	15	17.8	21	22
Glucose (mmol/L)	7.5	6.2	6.5	6.5	5.2	5.2	3.1	6.3

*ED*, emergency department; *CPB*, cardiopulmonary bypass; *ICU*, intensive care unit.
